# Supplementary material for: The interactome of intact mitochondria by cross-linking mass spectrometry provides evidence for coexisting respiratory supercomplexes
Source: Mol Cell Proteomics. 2017 Dec 8;17(2):216–32. doi: 10.1074/mcp.RA117.000470 (PMC5795388; doi:10.1074/mcp.RA117.000470)
Supplement: Supplemental Data [file supp_RA117.000470_133922_0_supp_23978_fzffwf.zip › spectra_annotation/mito_DR_spectra_annotation/380-1-5-1-11-1.pdf]

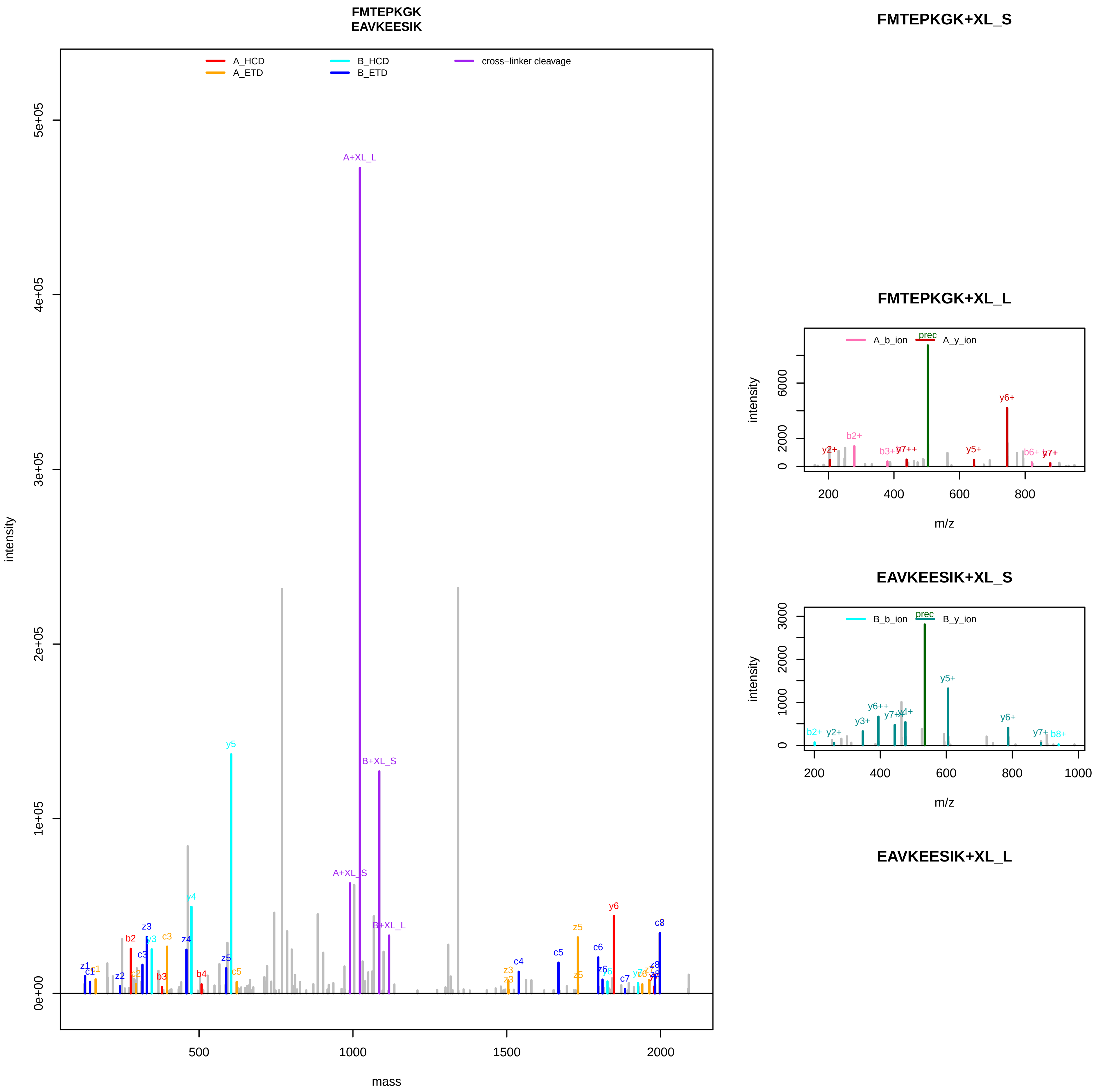
FMTEPKGK
EAVKEESIK
A_HCD
A_ETD
B_HCD
B_ETD
cross−linker cleavage
A+XL_L
B+XL_S
A+XL_S
B+XL_L
y5
y4
y6
z5
c8
c6
c5
c4
z3
z4
c3
b2
b3
b4
c5
c1
z1
z2
y3
intensity
mass
0e+00
1e+05
2e+05
3e+05
4e+05
5e+05
500
1000
1500
2000
FMTEPKGK+XL_S
FMTEPKGK+XL_L
A_b_ion
A_y_ion
prec
y6+
y5+
b2+
y2+
b3+
y7++
b6+
y7+
intensity
m/z
EAVKEESIK+XL_S
B_b_ion
B_y_ion
y5+
y6++
y7++
y4+
y3+
y6+
b2+
y2+
y7+
b8+
EAVKEESIK+XL_L
